# Acquisition of anoikis resistance in human osteosarcoma cells does not alter sensitivity to chemotherapeutic agents

**DOI:** 10.1186/1471-2407-5-39

**Published:** 2005-04-13

**Authors:** C Marcela Díaz-Montero, Bradley W McIntyre

**Affiliations:** 1 Department of Immunology, The University of Texas M. D. Anderson Cancer Center, Houston, TX, USA

## Abstract

**Background:**

Chemotherapy-induced cell death can involve the induction of apoptosis. Thus, aberrant function of the pathways involved might result in chemoresistance. Since cell adhesion to the extracellular matrix acts as a survival factor that homeostatically maintains normal tissue architecture, it was tested whether acquisition of resistance to deadhesion-induced apoptosis (anoikis) in human osteosarcoma would result in resistance to chemotherapy.

**Methods:**

Osteosarcoma cell lines (SAOS-2 and TE-85) obtained from ATCC and were maintained in complete Eagle's MEM medium. Suspension culture was established by placing cells in tissue culture wells coated with poly-HEMA. Cell cytotoxicity was determined using a live/dead cytotoxicity assay. Cell cycle/apoptosis analyses were performed using propidium iodide (PI) staining with subsequent FACS analysis. Apoptosis was also assayed by Annexin-FITC/PI staining.

**Results:**

Etoposide, adriamycin, vinblastine, cisplatin and paclitaxel were able to induce apoptosis in human osteosarcoma cells SAOS-2 regardless of their anoikis resistance phenotype or the culture conditions (adhered vs. suspended). Moreover, suspended anoikis resistant TE-85 cells (TE-85ar) retained their sensitivity to chemotherapy as well.

**Conclusion:**

Acquisition of anoikis resistance in human osteosarcoma cells does not result in a generalized resistance to all apoptotic stimuli, including chemotherapy. Moreover, our results suggest that the pathways regulating anoikis resistance and chemotherapy resistance might involve the action of different mediators.

## Background

During normal organ development apoptosis provides an efficient mechanism whereby unwanted cells are "discretely" eliminated. The functions that ensure proper activation of apoptosis are somehow lost during tumorigenesis, allowing cancer cells to proliferate indefinitely and in an uncontrolled fashion[1,2].

Apoptosis is triggered by several stimuli including hypoxia, radiation-induced DNA damage, oxidative stress, and lack of attachment. The term anoikis defines the type of apoptosis induced after proper adherence to the extracellular matrix (ECM) is denied[3,4]. Attachment to the ECM is mainly mediated by integrins; a family of heterodimeric transmembrane receptors composed of an alpha and a beta chain. In response to physiological clues, bidirectional integrin signaling mediates cell differentiation, proliferation, homing, migration and survival[5,6]. Integrins lack kinase domains; they signal by associating in complexes with other mediators such as FAK, ILK, Src, Shc, Syk, and paxillin [7-13]. Anoikis resistant tumor cells have circumvented the death signals generated by the lack of attachment affording them increased survival times while migrating to secondary sites. Thus resistance to anoikis has been regarded as a crucial step during tumorigenesis [14-16]. Our previous work has shown that human osteosarcoma cells, SAOS, are sensitive to anoikis[17]. However, anoikis resistance can be driven in originally sensitive clones by alternating culture cycles under adhered and suspended conditions. This resistant phenotype is stable and indicates that the processes of de-adhesion or exposure to a non-adhesive environment acts as a driving force towards anoikis resistance[18].

While the precise role of anoikis resistance in osteosarcoma progression is still unclear, chemoresistance continues to be an important problem in the clinic. Despite significant advances in the treatment of osteosarcoma, the prognosis of patients with metastasis at presentation remains poor, with an overall survival of 55% after aggressive chemotherapy and surgery[19,20]. Historically, resistance to chemotherapy has been attributed to the overexpression of genes encoding cellular efflux pumps[21]. Recent studies have shown that the action of many anti-cancer agents results in apoptosis, therefore alterations in the apoptotic pathway may also confer multidrug resistance [22-24]. Since the general acquisition of apoptosis resistance would affect both de-adhesion and chemotherapy-induced cell death, we investigated whether acquisition of anoikis resistance conferred general resistance to other apoptotic inducers or was independent of these other apoptotic pathways.

## Methods

### Cell culture and reagents

The parental human osteosarcoma cell lines SAOS-2 (SAOSp) and TE-85 (TE-85p) were obtained from the American Type Culture Collection (Manassas, VA). SAOSp and TE-85p cells were maintained in Eagle's MEM (BioWhittaker, Walkersville, MD), supplemented with 10% fetal bovine serum (BioWhittaker, Walkersville, MD), 2 mM L-glutamine, 1 mM sodium pyruvate and non-essential amino acids (Sigma, St. Louis, MO). Anoikis resistant SAOS (SAOSar) and TE-85 (TE-85ar) cells were generated by sequential cycles of culture on untreated (adhered) and poly-HEMA treated cell culture wells (suspended)[18]. The resulting variants were maintained in culture under adhered conditions. Poly-HEMA was prepared by dissolving it in 95% ethanol to a concentration of 50 mg/ml. Poly-HEMA was added to cell culture wells at a density of 5 mg/cm^2 ^and allowed to dry overnight, under sterile conditions in a laminar flow hood. Etoposide, vinblastine and paclitaxel were purchased from Sigma, St. Louis, MO. Cisplatin was purchased from Bristol-Myers Squibb Company, Princeton, NJ. Adriamycin was purchased from GensiaSicor™ Pharmaceuticals, Irvine, CA. *In vitro *LD_50 _for each agent was determined. One *in vitro *LD_50 _is defined as the dose required to induce apoptosis in approximately 50% of the cells in 24 hr of culture.

### Live/Dead cytotoxicity assay

SAOSp and SAOSar were cultured under suspended conditions (poly-HEMA treated cell culture wells) for 24 h. After culture cells were washed with PBS, and pellets resuspended in 250 μl of PBS containing 2 μM Calcein-AM and 8 μM ethidium homodimer-1 (Molecular Probes, Eugene, OR). Cells were incubated at room temperature for 15 minutes and visualized using a fluorescent inverted microscope.

### Apoptosis analyses

Cell cycle/apoptosis analyses were performed using propidiumiodide (PI) staining with subsequent FACS analysis. 5 × 10^5 ^cells/well were cultured either on plastic or poly-HEMA treated 6-well tissue culture plates with or without the metabolic inhibitors and drugs for 24 hrs at 37°C in a 5% CO2 atmosphere. After incubation, adherent cells were detached with trypsin (0.5% trypsin/0.1% EDTA in PBS). Detached and suspended cells were harvested in complete EMEM medium and centrifuged at 500 g for 10 min. Pellets were washed with PBS and fixed with ice cold 75% ethanol overnight at 4°C. After fixation, cells were washed with PBS and stained with 500 μl of PI solution (50 μg/ml in PBS) containing 25 μg/ml of RNase. Cells were incubated at 37°C for 30 min and analyzed by flow cytometry on an Epics Profile flow cytometer (Coulter, Miami, FL). Apoptosis was also assayed by Annexin-FITC/PI staining following manufacturer instructions (Trevigen, Inc. Gaithersburg, MD). Briefly, treated or untreated cells were collected and washed in cold PBS. Cells were incubated for 15 min at room temperature in the presence of 1 μl Annexin V-FITC, 1 μl of propidium iodide and 98 μl of 1x binding buffer (all reagents provided by the manufacturer). After incubation, 400 μl of 1X binding buffer was added to each tube, and cells were analyzed by flow cytometry.

## Results

**Figure 1:**
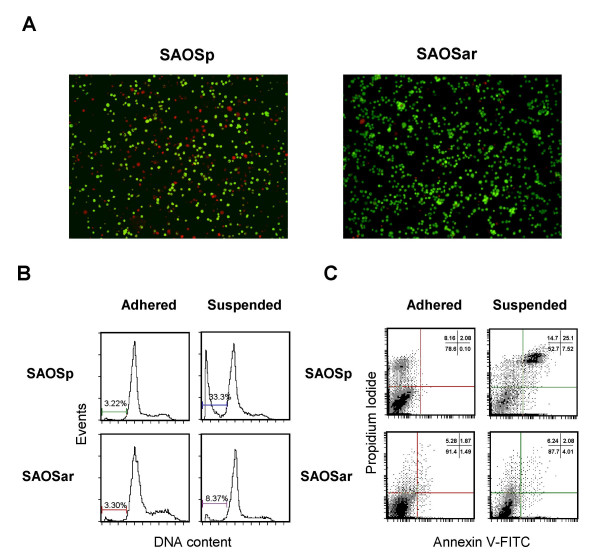
**SAOSp cells undergo anoikis after culture under suspended conditions. **After culture under suspended conditions for 24 h SAOSp and SAOSar cell viability was assayed using a Live/Dead™ assay. Panel A shows a larger fraction of SAOSp cells stained red (dead) than of SAOSar cells (majority stained green). Apoptosis was quantitated by PI (panel B) or Annexin V-FITC/PI (panel C) staining followed by flow cytometry analyses. Panel B shows a higher percentage of SAOSp (33.3%) cells than of SAOSar (8.37%) in the sub-G/0 phase representative of apoptotic cells. Similar results are shown in panel C, in which a higher percentage of SAOSp cells (32.6%) than of SAOSar (6.09%) stained positive for Annexin V (top and bottom right quadrants) representative of apoptotic cells. Results shown are representative of three independent experiments.

Parental human osteosarcoma SAOS-2 cells (SAOSp) undergo apoptosis after adherence to the ECM is denied (anoikis) by culture in poly-HEMA treated cell culture wells. An anoikis resistant subline (SAOSar) has been generated after sequential cycles of culture under suspended and adhered conditions. This stable phenotype is not the result of mere selection of pre-existing anoikis resistant sub-populations, since anoikis resistant cells can be derived from anoikis sensitive clonal populations[18]. Fig 1 panel A shows the results of a Live/Dead™ assay of SAOSp and SAOSar cells cultured in poly-HEMA coated wells for 24 hr. After incubation, cells were stained with a mixture of calcein-AM and ethidium homodimer-1 (see Materials section). Intracellular esterases in live cells convert non-fluorescent cell-permeant calcein-AM to green fluorescent calcein. By contrast, in non-viable cells with damaged membranes, non-permeant ethidium homodimer-1 enters the cells, and by binding to nucleic acids the ethidium homodimer-1 produces a bright red fluorescence. Cells were viewed under a fluorescent inverted microscope using a longpass filter in order to simultaneously visualize both green and red fluorescence. Panel A shows a larger fraction of the parental SAOSp cells stained red (dead), in comparison with the anoikis resistant SAOSar cells in which the majority of the cells stained green (live). Under control adherent conditions both SAOS populations were uniformly alive and stained green (data not shown). For the quantitative analysis of apoptosis, two assays were used, cell cycle analysis and a membrane quality assay. Cell cycle was analyzed by staining DNA with PI and determining by flow cytometry the percentage cells with sub-G/0 content. The membrane quality was assessed by utilizing Annexin staining to identify phosphatidylserine and PI to monitor membrane integrity. Figure 1B shows a higher percentage of SAOSp cells in sub-G/0 phase, indicative of apoptosis (33.3%) than of SAOSar cells (8.37%) after culture in poly-HEMA treated plates for 24 h. The same was observed after staining with Annexin V-FITC/PI and flow cytometry. Figure 1C shows higher percentage of SAOSp cells (32.6%) than of SAOSar cells (6.09%) stained positive for Annexin V (top and bottom right quadrants) representative of apoptosis as well. Thus, SAOSar cells resist apoptosis after attachment to ECM is denied (anoikis).

**Figure 2:**
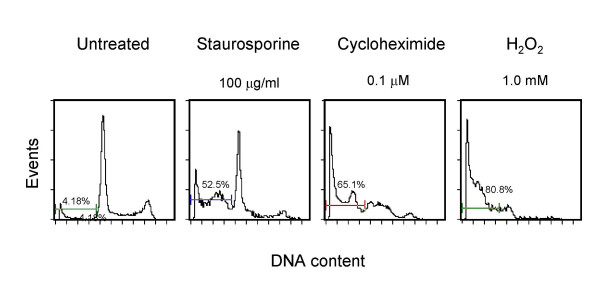
**Apoptosis can be induced in SAOSar cells while cultured under suspended conditions. **Apoptosis was triggered in suspended anoikis-resistant SAOSar cells by staurosporine-induced inhibition of calcium-dependent protein kinases, by cycloheximide-induced inhibition of protein synthesis and by H_2_O_2_-induced oxidative damage. Percentage apoptosis was determined by flow cytometry analyzes after staining with propidium iodide. The percentage of cells in the sub-G/0 phase representative of apoptotic cells is marked on each histogram. Results shown are representative of three independent experiments.

We have previously shown that SAOSp and SAOSar cells attached to the ECM are equally sensitive to induction of apoptosis and die after treatment with staurosporine, cycloheximide and hydrogen peroxide[18]. We hypothesized that even though the apoptotic machinery was intact while cultured under adhered conditions, once the anoikis resistant SAOSar cells were detached from the ECM, anti-apoptotic mediators could be activated resulting in a more generalized resistance to apoptosis. Therefore, to reexamine the sensitivity of anoikis resistant cells to other apoptotic stimuli, SAOSar cells were cultured under non-adherent conditions and exposed to staurosporine, cycloheximide or hydrogen peroxide. As shown in Fig. 2, untreated SAOSar cells are resistant to apoptosis when placed in nonadherent conditions, whereas staurosporine, cycloheximide, or hydrogen peroxide treatment of SAOSar cells results in apoptosis. These data suggest that the mechanisms conferring resistance to anoikis do not protect SAOSar cells from apoptosis induced by other stimuli.

**Figure 3:**
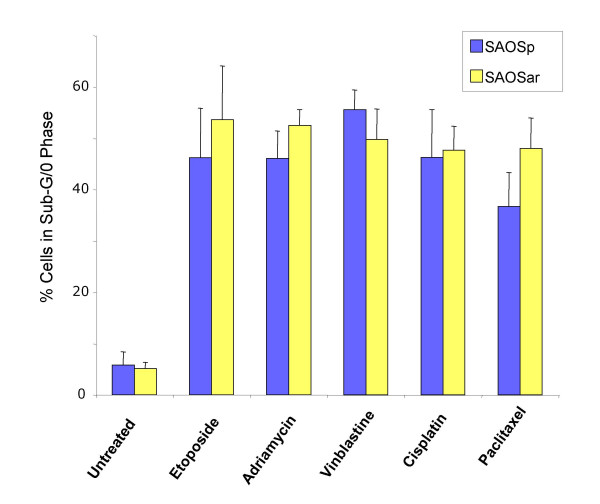
**Chemosensitivity of SAOSp and SAOSar cells. ***In vitro *LD_50 _for chemotherapy agents etoposide (50 μM), adriamycin (1 μg/ml), vinblastine (1 μg/ml), cisplatin (10 μg/ml) and paclitaxel (100 ug/ml) were determined for SAOSp and SAOSar cells cultured under adhered conditions. Similar chemosensitivity was observed for all agents tested by adhered SAOSp and SAOSar cells. Apoptosis was determined by PI staining followed by flow cytometry analyses. Error bars indicate the standard deviation of three independent experiments.

**Figure 4:**
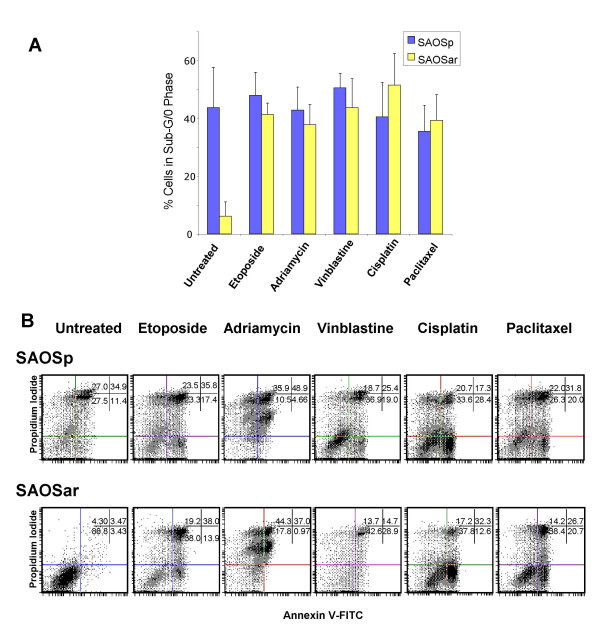
**Resistance to anoikis does not confer chemoresistance in SAOS cells. **Suspended SAOSp and SAOSar cells were treated with 1 *in vitro *LD_50 _of etoposide, adriamycin, vinblastine, cisplatin or paclitaxel. In Panel A, similar percentages of SAOSp or SAOSar cells in the sub-G/0 phase representative of apoptotic cells was found after incubation with the indicated agents for 24 h under suspended conditions. Error bars indicate the standard deviation of three independent experiments. Panel B shows similar percentages of Annexin V-FITC positive SAOSp or SAOSar suspended cells (top and bottom right quadrants) after the same treatments. Data shown is representative of three independent experiments.

Since the mechanism of action of certain chemotherapy agents can result in apoptosis we tested whether anoikis resistant SAOS cells were more resistant to chemotherapy-induced apoptosis. *In vitro *LD_50 _for chemotherapy agents etoposide, adriamycin, vinblastine, cisplatin and paclitaxel was determined for both SAOSp and SAOSar cultured under adhered conditions. Similar doses of the agents used were required to induce apoptosis in ≈ 50% of SAOSp and SAOSar cells cultured under adhered conditions for 24 h (Fig 3). We then tested whether culture under suspended conditions would have a chemoprotective effect in anoikis resistant SAOSar cells. SAOSp and SAOSar cells placed in suspended conditions (poly-HEMA coated cultured wells) were treated with *in vitro *LD_50 _of etoposide, adriamycin, vinblastine, cisplatin or paclitaxel. Apoptosis was assayed by PI and Annexin V-FITC/PI staining followed by flow cytometry analyses. Figure 4A shows that the percent of untreated SAOSp cells in the sub-G/0 phase representative of apoptosis is significantly increased in comparison to untreated SAOSar when cells are placed in suspension conditions. The chemotherapeutic agents do not appear to have an additive effect. By contrast, the untreated anoikis resistant SAOSar cells remain viable when placed in suspension but the cells retain their sensitivity to the chemotherapeutic agents and undergo apoptosis. This was corroborated by Annexin V-FITC/PI staining and flow cytometry analyses. Figure 4B shows a higher percentage of untreated SAOSp cells positive for Annexin V/FITC staining (top and bottom right quadrants) than of untreated SAOSar cells after culture under suspended conditions for 24 h. Chemotherapy-treated suspended SAOSp and SAOSar showed similar percentages of cells stained with Annexin V-FITC indicative of apoptosis.

**Figure 5:**
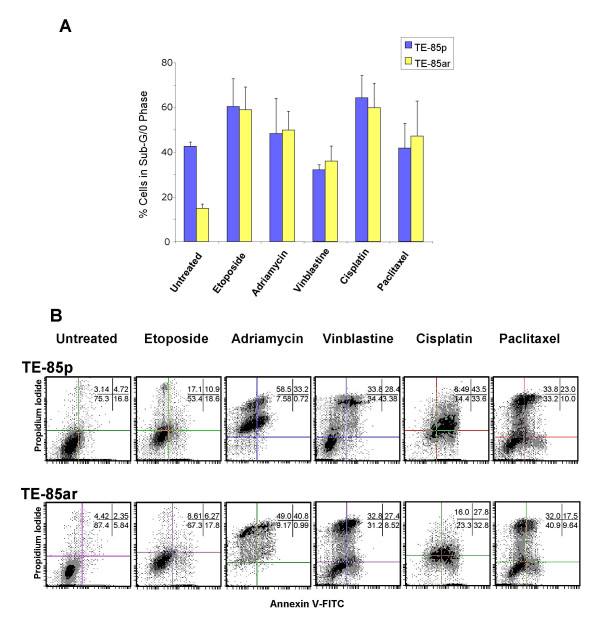
**Resistance to anoikis does not confer chemoresistance in TE-85 cells. **Suspended TE-85p and TE-85ar cells were treated with etoposide, adriamycin, vinblastine, cisplatin or paclitaxel for 24 h and apoptosis was assayed by PI (panel A) or Annexin V-FITC/PI (panel B) staining followed by flow cytometry analyses. In panel A similar percentages of TE-85p or TE-85ar cells in the sub-G/0 phase representative of apoptotic cells were found after incubation with the indicated agents. Error bars indicate the standard deviation of three independent experiments. Panel B shows similar percentages of Annexin V-FITC positive TE-85p or TE-85ar suspended cells (top and bottom right quadrants) after the same treatments. Data shown is representative of three independent experiments.

A second osteosarcoma cell line, TE-85, was used to determine if chemotherapy sensitivity despite anoikis resistance was unique to SAOS-2 cells or represented a more generalized phenomenon among osteosarcoma. Anoikis resistant TE-85 cells (TE-85ar) were generated following the same procedure used for generating the anoikis resistant SAOSar cells. Suspended anoikis sensitive (TE-85p) and anoikis resistant (TE-85ar) cells were treated with the same doses of the same drugs and apoptosis was measured 24 hr later by PI and Annexin V-FITC/PI staining followed by flow cytometry analyses. As with SAOS-2 cells, untreated TE-85ar cells remained viable after culture in suspension for 24 hr but retained their sensitivity to chemotherapy-induced apoptosis at similar levels that of TE-85p cells. No significant differences among the percentages of apoptotic TE-85p or TE-85ar suspended cells were found after chemotherapy treatment, either by PI (figure 5A) or by Annexin V-FITC/PI (figure 5B) staining. These results indicate that resistance to anoikis does not confer resistance to these chemotherapeutic agents, and that this trend is not unique to SAOS-2 osteosarcoma cells but also applies to TE-85 osteosarcoma cells.

## Discussion

During tumorigenesis the delicate balance between survival and cell death is altered. Thus, cancer cells are able to survive under adverse conditions that normally would trigger apoptosis such as hypoxia, low glucose, and lack of attachment.

Until recently it was thought that resistance to chemotherapy was due to mechanisms that prevented the intake of the drug or the presence of intracellular detoxificants. Since the discovery that drug-mediated cell death can be the result of physiological processes such as apoptosis, mitotic catastrophe and cellular senescence, resistance to chemotherapy has been linked to alterations in the pathways mediating such processes. [22-26].

Resistance to detachment-induced apoptosis (anoikis) is known as a important step during metastasis by affording tumor cells increased survival times while migrating to secondary sites. However, the relationship between anoikis resistance and chemotherapy response remains to be elucidated. Previously, we have shown that anoikis resistance can be induced in anoikis sensitive human osteosarcoma cells, SAOS-2, by exposure to culture in suspension (poly-HEMA treated culture wells). We also demonstrated that oxidative damage (H_2_O_2_), inhibition of protein synthesis (cycloheximide) or inhibition of calcium-dependent protein kinases (staurosporine) resulted in apoptosis of adherent SAOS-2 cells regardless of their anoikis resistant phenotype[18]. This suggested that under adhered conditions the apoptotic machinery was intact.

In this study, we tested whether the anti-apoptotic mechanisms that rendered the cells anoikis resistant would be activated upon detachment from the ECM, resulting in a more generalized resistance to apoptosis and hence to chemotherapy. For instance, in acute myelogeneous leukemia interactions between α4β1 integrins and fibronectin activate the PI3-K/Akt pathway resulting in resistance to both anoikis and to treatment with daunorubicin or AraC[27]. By contrast, our data suggested that despite the resistant phenotype and the suspended conditions, apoptosis can still be induced by oxidative damage, inhibition of protein synthesis or inhibition of calcium-dependent protein kinases in anoikis resistant SAOSar cells. Furthermore, anoikis resistant SAOSar cells are equally sensitive to chemotherapy-induced apoptosis when compared to anoikis sensitive SAOSp cells under either suspended or adhered culture conditions. Similar results were obtained after anoikis sensitive and anoikis resistant TE-85 cells were treated with the same agents.

The chemotherapeutic agents tested vary widely in their mode of action; etoposide, adriamycin and cisplatin cause DNA damage by forming DNA adducts or by inhibiting topoisomerase II resulting in DNA breaks. Vinblastine and paclitaxel target the microtubules and are known as "spindle poisons", however their mode of action is different. Vinblastine binds to tubulin dimers preventing the formation of microtubules and paclitaxel binds to the microtubules inducing mitotic arrest by excessively stabilizing them. Regardless of their mode of action, under adhered conditions the *in vitro *LD_50 _for etoposide, adriamycin, vinblastine, cisplatin or paclitaxel was similar for both SAOSp and SAOSar cells. Similar levels of apoptosis were found after suspended SAOSp and SAOSar cells were treated with the same doses of the different agents. The same was observed after suspended TE-85p and TE-85ar cells were treated with the same agents. These data suggest that acquisition of anoikis resistance does not necessarily render osteosarcoma cells resistant to other apoptotic stimuli including chemotherapy.

The specific mechanisms involved in anoikis resistance are not completely understood. Overexpression of oncogenes such as *ras*, *raf*, *rac *and *src *as well as the deletion of tumor suppressor genes such as *PTEN *and *p53 *have been associated with resistance to anoikis [28-30]. Recently it was reported that TrkB, a nerurotrophic tyrosine kinase receptor, is able to suppress anoikis of non-malignant epithelial cells by activating the PI3-K/Akt pathway[31]. Activated Akt exerts its anti-apoptotic effect by modulating the activity of mediators that are directly involved in the apoptotic cascade or by regulating the transcription of pro- and anti-apoptotic genes [32-34]. Normal breast epithelial cells expressing constitutively active Akt1 lose their sensitivity to anoikis and become resistant to apoptosis after treatment with cisplatin and mitoxantrone[35]. Likewise, in pancreatic adenocarcinoma cells increased activity of Akt in response to overexpression of carcinoembryionic antigen-related cell adhesion molecule (CEACAM)6 results in resistance to both anoikis and gemcitabine treatment[36]. In these systems, Akt protects cells against death induced after DNA damage as well as death induced by anoikis. In our osteosarcoma model, it is clear that resistance to anoikis is independent of resistance to other apoptotic inducers. We have recently found that activation of the PI3-K/Akt pathway is important during anoikis resistance (Díaz-Montero CM and McIntyre BW, unpublished data). Since these cells are still sensitive to other apoptosis inducing agents, upregulation of Akt is not sufficient to confer resistance to all apoptotic stimuli.

Furthermore, in breast cancer cell lines SKBR-3 and MDA-MB-453, anoikis resistance can be restored by induction of ILK, independently of Akt activity[37]. These different studies suggest that the mechanisms that confer resistance to anoikis and/or chemotherapy may be unique to each type of malignancy. Thus it can be argued that anti-cancer agents that target apoptosis will be less effective against malignancies in which the pathways for resistance to both apoptosis and anoikis overlap. Likewise, therapies that can inhibit a common apoptosis and anoikis resistance pathway could be a potent new anti-cancer treatment.

Our work suggests that in at least two human osteosarcoma cell lines, resistance to anoikis and apoptosis are regulated by different mediators, and might explain why anoikis resistant cells are still vulnerable to other apoptosis-inducing stimuli. This is a fortunate situation except in those cases where the tumor cells have detached from the primary sites and survive because of the induction of anoikis resistance. In many cases, tumor cells that lose normal adhesive constraints will enter cell cycle arrest and thus be refractory to many chemotherapeutic reagents. In this scenario, the ability to target the anoikis resistance pathway may provide a new approach for chemotherapy.

In conclusion, in order to effectively target anoikis resistant/migrating metastatic tumor cells the apoptotic pathways that are altered and the ones that remain normal need to be identified. Only then, agents with specific action against the mediators involved during the relevant disease stage, i.e. primary vs. metastatic, will become available as more efficient treatment strategies.

## Conclusion

The development of newer and more efficacious treatments against cancer will require the understanding of the mechanisms behind apoptosis and/or anoikis resistance in a disease-specific way. We have shown that acquisition of resistance to anoikis in human osteosarcoma cells does not necessarily result in a generalized resistance to all apoptotic stimuli. Thus in this particular system, targeting the pathways involved might control the spread of anoikis resistant/migrating metastatic cells.

## Competing interests

The author(s) declare that they have no competing interests.

## Authors' contributions

CMDM designed and executed the experiments, collected and analyzed the data and drafted the manuscript. BWM designed experiments, interpreted the results, and edited the manuscript. All authors read and approved the final manuscript.

## Pre-publication history

The pre-publication history for this paper can be accessed here:
